# Supplementary figures and images for: Evaluation of a novel point-of-care lateral flow assay screening for Neisseria gonorrhoeae infection among pregnant women in Zimbabwe
Source: PLOS Glob Public Health. 2025 Feb 11;5(2):e0003839. doi: 10.1371/journal.pgph.0003839 (PMC11813084; doi:10.1371/journal.pgph.0003839)

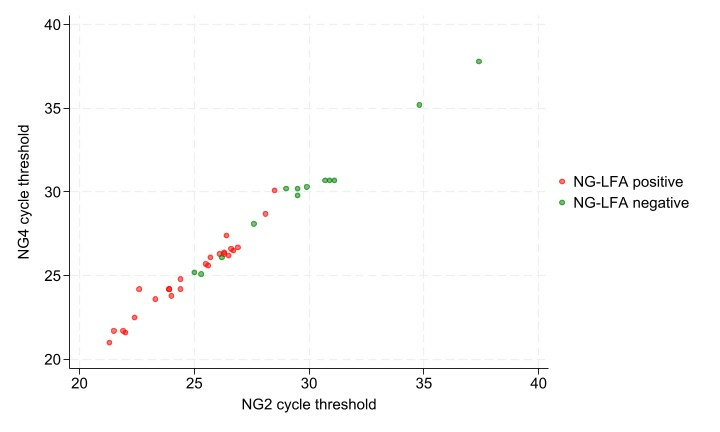

Supplement: S1 Fig — (TIF) [file pgph.0003839.s001.tif]
