# Supplementary material for: Evaluation of a novel point-of-care lateral flow assay screening for Neisseria gonorrhoeae infection among pregnant women in Zimbabwe
Source: PLOS Glob Public Health. 2025 Feb 11;5(2):e0003839. doi: 10.1371/journal.pgph.0003839 (PMC11813084; doi:10.1371/journal.pgph.0003839)
Supplement: S1 Table — (DOCX) [file pgph.0003839.s002.docx]

## Supplemental material B: Characteristics of participants positive for Neisseria gonorrhoeae on Xpert, comparing those with NG-LFA positive and negative results (N=38 unless otherwise stated)

|  | True positive (N = 25) | False negative (N = 13) | Total (N = 38) |
| --- | --- | --- | --- |
| **Vaginal discharge**  Present  Absent | 4 (16.0%)  21 (84.0%) | 3 (23.1%)  10 (76.9%) | 7 (18.4%)  31 (81.6%) |
| **Cycle threshold (NG2)**  <30  >30 | 25 (100.0%)  0 (0.0%) | 8 (61.5%)  5 (38.5%) | 33 (86.8%)  5 (13.2%) |
| **Cycle threshold (NG4)**  <30  >30 | 24 (96.0%)  1 (4.0%) | 5 (38.5%)  8 (61.5%) | 29 (76.3%)  9 (23.7%) |
| **Antibiotic usage in previous 2 weeks**  Yes  No | 0 (0.0%)  25 (100.0%) | 0 (0.0%)  13 (100.0%) | 0 (0.0%)  38 (100.0%) |
| **HIV status**  Positive  Negative | 5 (20.0%)  20 (80.0%) | 1 (7.7%)  12 (92.3%) | 6 (15.8%)  32 (84.2%) |
| **Age**  15 – 19  20 – 24  25 – 29  30 + | 9 (36.0%)  5 (20.0%)  4 (16.0%)  7 (28.0%) | 5 (38.5%)  2 (15.4%)  2 (15.4%)  4 (30.8%) | 14 (36.8%)  7 (18.4)  6 (15.8%)  11 (29.0%) |
| **Trimester (N=36)**  First  Second  Third | 1 (4.3%)  7 (30.4%)  15 (65.2%) | 1 (7.7%)  4 (30.8%)  8 (61.6%) | 2 (5.6%)  11 (30.6%)  23 (63.9%) |
