# Supplementary material for: Evaluation of a novel point-of-care lateral flow assay screening for Neisseria gonorrhoeae infection among pregnant women in Zimbabwe
Source: PLOS Glob Public Health. 2025 Feb 11;5(2):e0003839. doi: 10.1371/journal.pgph.0003839 (PMC11813084; doi:10.1371/journal.pgph.0003839)
Supplement: S2 Table — (DOCX) [file pgph.0003839.s003.docx]

## Supplemental material B: Themes and supporting quotes from interviews

| Theme | Supporting quotes |
| --- | --- |
| Ease of use and interpretation | “It is easy to use like even for interpretation you won’t face any challenges at all except when the results would come back as negative yet the GeneXpert result would be positive.” (nurse)  “It was easy and you just need to do the controls first.” (research assistant)  “It was easy to use compared to using the GeneXpert machine” (nurse)  “In interpreting the results there was no problem at all. It was quite easy to use” (nurse)  “I think we had everything in our fingertips. I don’t think I would constantly refer to them [flashcards detailing NG-LFA procedures] except for when the results came out discrepant; that’s when I would go back to check and see if I had missed some step or checking whether I had gone wrong somewhere just to see if it is correct or not” (54) |
| Positive device characteristics | “It was portable and very light and easily charged. It would have long-lasting battery as well. Like 30 minutes to have the results haa that’s wonderful” (research assistant)  “The size is portable and very easy to carry” (nurse) |
| Discrepancies with a seemingly infallible gold standard, leading to mistrust of results | “The only challenge like at first it looked like it’s getting the same result as the GeneXpert. It would pick but the GeneXpert wouldn’t pick and then at the end it would not pick at all. I don’t know whether it’s calibration or what exactly will be happening. So, I don’t know where the discrepancy really was and what caused it but it was like that.” (nurse)  “The FIND machine (NG-LFA) was okay and very simple to follow. It was easier to use and it is very user-friendly only that on the readings I didn’t really love the results. Because you would find that if the flow test indicated a negative test, the GeneXpert machine would reveal a positive result of the same sample used. So, there were some discrepancies with its results.” (research assistant)  “Although the flow test would record false negatives or false positives at times like when the GeneXpert machine results came out they would be different from the flow test results” (research assistant)  “The results on the flow test were not reliable.” (research assistant) |
| Importance of a back-up test during future use | “Well, I think it is better to use it as a second reader. Plus, for tests, people say that it is better for a test to detect something than for people to guess the results. If it doesn’t detect and maybe the person’s symptoms are not going away yet the result is a negative one then maybe the person will be treated. So, I think it helps in assisting to know if a true result is indeed a true result by being used alongside another detector like GeneXpert machine.” (nurse) |
