# Supplementary material for: Evaluation of a novel point-of-care lateral flow assay screening for Neisseria gonorrhoeae infection among pregnant women in Zimbabwe
Source: PLOS Glob Public Health. 2025 Feb 11;5(2):e0003839. doi: 10.1371/journal.pgph.0003839 (PMC11813084; doi:10.1371/journal.pgph.0003839)
Supplement: Attachment — Submitted filename: NG-LFA_rebuttal_25072024.docx [file pgph.0003839.s005.docx]

25^th^ July 2024

Dear Dr Kyobutungi and Professor Pai,

Thank you for considering our manuscript (PGPH-D-24-01660) for publication in *PLOS Global Public Health*. We initially submitted the article to *PLOS Medicine*, for which the editorial and reviewer comments and our responses are detailed below.

Comments from the Academic Editor:

1) I think reporting could be clearer. I appreciate this evaluation was nested within a larger study, but details of other testing procedures and tests make for a somewhat distracting read. I would advise the authors to frame as a diagnostic accuracy evaluation of the new NG lateral flow test only, and omit unnecessary other study details. Potentially, the alternative way to approach this would be that there is a single study protocol that evaluates multiple different tests and outcomes. Would the authors be amenable to publishing a consolidated report describing all tests and outcomes (including patient-level and birth outcomes) as specified in the protocol?

Thank you for your comments. We have tried to strike a balance between including sufficient information from the parent study for readers to understand the context of the study without being overly burdensome. We are reticent to remove details of the other tests performed (in paragraph one of the “study procedures” section of the methods) as this provides crucial information on the order of sampling, which we now discuss as a potential reason for low bacterial loads, as follows:

*“Another consideration that may have contributed to lower bacterial loads was that the NG-LFA specimen was the third swab collected. In contrast, in the previous evaluation by Peters et al. the first swab collected was tested using the NG-LFA.[10]” [page 13, lines 430-432]*

Other than this, it is not entirely clear what other study details would be suggested to be omitted.

Unfortunately, we would not consider publishing such a consolidated report describing all tests and outcomes, as the objectives of the parent study are very diverse and largely focussed on implementation. Any such paper would therefore be very cumbersome and unfocussed.

2) Needs to be fully reported in line with STARD guidelines (e.g. currently has a "CONSORT" diagram as figure 1) - emphasis should be on comparison the index test and reference standard. Currently, despite a STARD checklist being uploaded, not all items are fully reported.

Reference to the “CONSORT” diagram has been removed, and replaced with “flow diagram” (concurrent with STARD guidelines).

The following items of the STARD checklist were originally recorded as not reported, and have now been amended:

12b. Definition of and rationale for test positivity cut-offs or result categories of the reference standard, distinguishing pre-specified from exploratory

*“Cycle threshold values for Xpert-positive results were extracted from the GeneXpert output for both NG2 and NG4 targets, which are two separate chromosomal targets for NG, that are amplified as part of results processing [18]. Both NG2 and NG4 targets must be detected for the Xpert result to be reported as positive.” [page 5, lines 180-183]*

17. Any analyses of variability in diagnostic accuracy, distinguishing pre-specified from exploratory

*“Exploratory descriptive analyses were conducted to assess these measures according to symptom status, polymerase chain reaction (PCR) cycle threshold (Ct) value, antibiotic usage, HIV status, age, trimester, clinic site, and NG-LFA reader.” [page 6, lines 225-227]*

22. Time interval and any clinical interventions between index test and reference standard

This was already reported in the methods, as it was clear that both index test and reference standard were performed on the same day.

25. Any adverse events from performing the index test or the reference standard

*“No adverse events were reported from performing either the NG-LFA or Xpert.” [page 11, line 345]*

The following items on the STARD checklist are still recorded as “N/A” as we do not believe they are relevant for the present study, with justification provided:

21b. Distribution of alternative diagnoses in those without the target condition

As a screening study, for those without the target condition (in this case NG infection), they did not necessarily have any alternative diagnoses.

28. Registration number and name of registry

Although we registered the main parent study (NCT05541081) with ClinicalTrials.gov, we did not pre-register the current diagnostic accuracy study of the NG-LFA.

3) I was confused by the reference standard for statistical comparison with the index test (Xpert molecular testing). NG culture was also done, and there seems to be considerable discrepancy between the Xpert and culture results, which is not really addressed by the authors. The authors make reference to a study protocol being previously published, which I have reviewed, but there is very little detail about this diagnostic accuracy evaluation being reported here. Specifically, there is no mention of the index test or reference standards for this comparison. Further sample sizes are powered around associations between risk factors and prevalence of infection. Overall, I think it is a bit of a stretch to say that there is a protocol for this diagnostic accuracy evaluation.

As per the previous evaluation by Peters et al., the reference standard was Xpert molecular testing. As is well documented, sensitivity of gonococcal culture (particularly using cervical swabs from asymptomatic women) is lower than for molecular testing. We do note that sensitivity of culture was particularly poor in our study. However, it should be noted that we did not have ideal conditions for gonococcal culture. We did not have access to an on-site incubator so could not plate the swabs immediately onto agar. Furthermore, due to logistic constraints, although collected on the same day as sample collection, there was often several hours between sampling and transport to the laboratory for plating. We have now included the following sentences in the limitations section to acknowledge this:

*“Unfortunately, overall yield from culture of NG isolates was relatively poor, with just over a third of individuals with Xpert-positive NG having a positive culture result. Although NG culture was never intended to be the gold standard, it was still less helpful than had been envisioned, particularly in terms of confirming false positive or false negative results. The lack of an on-site incubator to allow for immediate plating, and also variable times between sample collection and transport to the laboratory may have been contributing factors.” [page 15, lines 514-519]*

Regarding reference to the protocol, at no point in the manuscript is it stated that “there is a protocol for this diagnostic accuracy evaluation”. In relation to the protocol, we stated:

*“The parent study protocol has been published.” [page 4, lines 134-135]*

*“Sample size was calculated based on the parent study.” [page 5, lines 220-221]*

To emphasise this, in the limitations section of the discussion we now also state:

*“Importantly, the overall study sample size was not powered for the present evaluation and was likely underpowered.” [page 15, lines 507-508]*

4) Building on point 3 above, the low sensitivity of the NG LF test, and very wide uncertainty means that the study was likely underpowered for this diagnostic accuracy comparison. We can see this in table 3 of the protocol paper, where the sample size of 896 women is selected based on an assumed prevalence of STI of 20-30%. But, in this report, prevalence of Xpert positive NG was only 4.2%.

We have already stated in the limitations section: *“As the number of women with positive Xpert results was relatively small, the confidence intervals for the sensitivity estimates are wide, limiting comparisons of test performance across different participant characteristics.” [page 15, lines 505-507]*

We have now specifically commented on the sample size in the limitations section, as follows:

*“Importantly, the overall study sample size was not powered for the present evaluation and was likely underpowered.” [page 15, lines 507-508]*

5) I am unconvinced by the stratified diagnostic accuracy estimates (Table 3) - numbers are extremely small for all comparisons, and I think the authors are over-interpreting here.

Thank you for your comment. We are aware of the limitations of making comparisons across groups, which is why we have already stated this twice in the discussion section, as follows:

*“We compared sensitivity and specificity across a range of participant characteristics. However, due to the small sample sizes for sensitivity calculations, it is difficult to draw meaningful conclusions from these observations.” [page 14, lines 459-461]*

*“As the number of women with positive Xpert results was relatively small, the confidence intervals for the sensitivity estimates are wide, limiting comparisons of test performance across different participant characteristics.” [page 15, lines 505-507]*

Furthermore, we have purposefully not made any such comparisons in the discussion section. We appreciate that there is an argument to remove such disaggregated data entirely from the manuscript. However, we feel it does provides helpful additional context. We have however decided to move table 3 to the supplemental material.

6) Likewise I don't think the qualitative results add much to our understanding of why sensitivity was so much lower than the previous study - is insufficiently detailed or rich to provide much meaningful insights.

The aim of the qualitative component of the study was not to assess why sensitivity was lower than in the previous study. As stated in the methods, interviews focussed on acceptability and usability. This has also previously been explored by de Vos et al. (2023) when testing symptomatic individuals in South Africa. Our study adds to the literature by providing additional insight from intervention team members involved in conducting testing using the NG-LFA, but as a screening tool. Crucially, we were able to compare and contrast some of the findings with the study by de Vos et al, and discussed why there were differences in perceptions of the NG-LFA. We acknowledge that the amount of qualitative data was relatively limited – however, we feel it is a useful accompaniment to the main accuracy data.

7) Disagree with the main conclusion that this test is "promising" based on the results presented here. Sensitivity (as reported here) is too low for a screening test to be used in asymptomatic women. I would have liked to have seen much greater interrogation about potential reasons for low sensitivity (index test methods, reference standard used, under-powered?).

The full sentence reads: *“In conclusion, although the NG-LFA is promising for augmenting syndromic management in order to prevent inappropriate antimicrobial usage,[10] the potential usability of the NG-LFA as a screening tool in this context is less evident.”*

As is clear from the full sentence (and the associated reference), the NG-LFA is promising for its use in symptomatic individuals, as demonstrated by Peters et al. in South Africa. We clearly state “potential usability” in the our context (screening in ANC) is “less evident”. To make this more clear, the sentence has been amended as follows:

*“In conclusion, although the NG-LFA is promising for augmenting syndromic management in order to prevent inappropriate antimicrobial usage, as demonstrated among symptomatic individuals in South Africa,[10] the potential usability of the NG-LFA as a screening tool in this context is less evident.” [page 15, lines 528-530]*

We already discuss potential reasons for false negative results, including low bacterial load, human error and issues with the readers. Furthermore, we discuss in detail how a molecular reference standard may detect prior or resolving infections which may self-clear, the difference between antigen tests and NAATs, and that Xpert is not a perfect reference diagnostic. It is therefore unclear what additional information regarding “the reference standard used” would be helpful.

Regarding index test methods, we have added the following sentence, discussing how sampling order may have affected sensitivity:

*“Another consideration that may have contributed to lower bacterial loads was that the NG-LFA specimen was the third swab collected. In contrast, in the previous evaluation by Peters et al. the first swab collected was tested using the NG-LFA.[10]” [page 13, lines 430-432]*

We have also added additional analyses with the production of a ROC curve, and a new paragraph in the discussion focussed on how the assigned fluorescence threshold for the NG-LFA may have influenced sensitivity and specificity.

The limitations section now states that the study was likely underpowered, as follows:

*“Importantly, the overall study sample size was not powered for the present evaluation, and was likely underpowered.” [page 15, lines 507-508]*

8) Finally, I have reviewed the abstract of the Lancet paper, which was done among symptomatic individuals, and so we would expect higher sensitivity. In this paper, the authors would need to provide much greater discussion about why the sensitivity they found was so much lower than Peters' et al.

As discussed in response to point number seven, we discuss several potential reasons for low sensitivity of the NG-LFA as a screening tool. As stated in the discussion we are wary about focussing too much on comparisons with the Peters et al. study. We state that:

*“Caution must also be exercised when comparing these findings to the study among symptomatic individuals in South Africa.[10] Individuals attending clinic because they have symptoms are likely very different to those attending for routine ANC and reporting symptoms when specifically asked. Firstly, physiological changes to vaginal discharge are common in pregnancy. Furthermore, the former’s symptoms are more likely to be of a higher severity or duration such that the individual has sought care.” [page 14, lines 461-466]*

However, as mentioned above, we have added an additional paragraph focussed on fluroresence thresholds and how a particular threshold may be suitable for one population (i.e. symptomatic patients) but not another (i.e. ANC).

Reviewer #1:

This article is on the use of a lateral flow essay for Neisseria gonorrhoeae in a Zimbabwean population. While the aims and methods are generally clear to follow and the analysis approach seems broadly reasonable, I believe there are nonetheless some issues that aren't addressed within the current submission.

The main issue is that the paper largely follows similar themes to the Lancet article which stated the accuracy of the NG-LFA in a South Africa population (reference 10). The key difference though appears to be that in the Lancet paper symptomatic participants were included for confirmation of diseases status, whereas in this study asymptomatic individuals were included. The disease prevalence among tested individuals will therefore be much lower compared to the Lancet study. The aim of this submission is that the performance of the NG-LFA is to be assessed as a screening tool and that this is a novelty not part of the study involved in the Lancet submission. However, supplementary table S1 of the Lancet paper indicates clearly why this was not considered appropriate, as with a low prevalence of disease the NPV and PPV values will be considerably worse than if symptomatic individuals were being assessed. Indeed the values presented in this submission (4.2% prevalence, 78.1% PPV, 98.5% NPV) are broadly in line with the hypothesised values in the Lancet table S1 at 5% prevalence (56.6% PPV and 99.6% NPV).

In short, it is not surprising that the NG-LFA is ineffective as a screening tool as this was not the intended purpose since with low prevalence the PPV will by definition be considerably lower.

Thank you for your comments. We agree that we would hypothesise that PPV would be lower in asymptomatic populations, stating in the introduction “Clinical test performance may differ if bacterial loads are lower among asymptomatic than symptomatic individuals whilst the predictive values for both negative and positive results will be affected by the prevalence among different populations.” However, that should not preclude conducting such evaluations to ensure that is the case, particularly given the very high sensitivity and specificity demonstrated in the evaluation in symptomatic individuals. In fact, the article detailing the development of the NG-LFA (Gleeson et al; March 2024; DOI: 10.1097/OLQ.0000000000001913), specifically states “Based on the preliminary performance assessed by these studies, future work should advance to in-country field evaluations in LMICs for asymptomatic populations and other specimen types as noted previously. Ongoing field evaluation is a critical step in supporting the development of these technologies to meet the needs of LMICs.”

There are some other details that I believe are currently missing or unclear:

- If the NG-LFA was to be attempted to use as a screening tool, the thresholds previously established ("Fluorescence threshold for positive reading is ≥0·063") to use for confirming diagnosis on symptomatic individuals may no longer apply but there doesn't seem any analysis or discussion on this. For example it may be possible to show that a change in threshold could be applied that would sacrifice some of the specificity for increased sensitivity that could be more useful as a diagnostic tool.

- No ROC curves or AUC summaries are included but could be useful as supplementary information.

Thank you for your very helpful comments. We have now made it clear that the fluorescence threshold for a positive reading was also ≥0·063 in the methods, as follows:

*“Fluorescence is detected by a small portable point-of-care reader that classifies results into positive, negative, or invalid, within 20 minutes using simple LED light indicators. The fluorescence threshold for a positive reading was ≥0·063.” [page 4, lines 160-163]*

We have also generated a ROC curve, and calculated the AUC and Youden index, detailed in the methods as follows:

*“A receiver operating characteristic (ROC) curve, and the area under the curve, was generated from the NG-LFA fluorescence values, with Xpert as the reference standard. The Youden index was also calculated, using the “cutpt” command in Stata, which is the value of the threshold which maximises both the sensitivity and specificity of a test, giving equal weight to each.” [page 6, lines 231-234]*

The following information on ROC curve and AUC are now included in the results:

*“The ROC curve is shown in figure 3. The AUC was 0.94 (95% CI 0.89 – 0.98). The optimal cut point according to the Youden Index was ≥0.0278785, which provided a sensitivity, specificity, PPV, and NPV, at this cut point of 89.5% (95% CI 75.2 – 97.1%), 90.4% (95% CI 88.2 – 92.2%), 28.8% (95% CI 20.8 – 37.9%), and 99.5% (95% ci 98.7 – 99.9%), respectively.” [page 12, lines 373-376]*

We also added the following paragraph to the discussion:

*“ Such differences between populations are also important when considering the fluorescence threshold at which a sample is considered NG positive, and how optimal thresholds will vary between different populations. In both this study and the evaluation among symptomatic patients in South Africa, the threshold for a positive reading was ≥0.063. However, in our study a reduced threshold of ≥0.0278785 would have led to a sensitivity and specificity of 89.5% and 90.4%, respectively. By sacrificing a degree of specificity, only 4 NG infections out of 38 would have been missed. However, one in ten of those without NG would have been inappropriately treated. This demonstrates the importance of the aim of screening or testing in determining the most appropriate balance between sensitivity and specificity. For example, if the primary goal of ANC NG screening is to avert cases of ophthalmia neonatorum, then a relatively high rate of overtreatment may be tolerated. Finally we calculated the Youden index, which gives equal weight to sensitivity and specificity.[20] However, a different weighting may be used to find the most appropriate threshold. Importantly, as the NG-LFA has not yet gone through regulatory approval and is not commercialised, the threshold in both this study and the evaluation among symptomatic individuals was based on limited data. The threshold for a commercialised assay would be based on larger datasets covering different populations.” [page 14, lines 468-482]*

- Line 401-403 states that "Based on the findings of this study the NG-LFA provides value in this context by allowing identification of asymptomatic women with NG who would otherwise be untreated, and may serve as a useful screening tool to rule in and treat NG infection in pregnant populations." I am doubtful of this though, as without confirmation of symptoms or the Xpert results then it may be that the identification of these are false positives.

Although confirmation would be ideal, it is important to consider the settings in which this test was evaluated and would potentially be embedded. At present, the current standard of care is no screening, with treatment for gonorrhoea only provided if symptoms are present. Syndromic management of vaginal discharge will lead to treatment of many women who do not have gonorrhoea, while also missing all asymptomatic gonococcal infections. Therefore, having an NG-LFA that can detect and treat infections that would otherwise be untreated may potentially be useful, particularly with regards to preventing ophthalmia neonatorum.

To qualify our statement, we have now added the word “potentially” before “provides value in this context…”

- It is stated in analysis section that "Sample size was calculated based on the parent study.". While this may be true in that are constrained in sample size by the parent study, this then  doesn't provide any information on the power of the study in advance of collecting data.

We have now emphasised this in the limitations section, as follows:

*“Importantly, the overall study sample size was not powered for the present evaluation, and was likely underpowered.” [page 15, lines 507-508]*

Reviewer #2: The text is very well written and understandable. The results show that there will be a long way to improve the test and give it a better sensitivity for asymptomatic women. In my view the most vulnerable, and without symptoms and no regular screening have mor consequences in their sexual and reproductive health.

Thank you for your comments.

In the paper a detail caught my attention, on line 172 the authors wrote "For participants positive for NG on either Xpert or the NG-LFA, an additional cervical swab was collected for gonococcal culture". Considering the participants of the study were pregnant women, I think it would be necessary to mention some additional consideration about the wellbeing of the foetuses, since cervical sample collection in pregnant women implies risk.

Thank you for your comments. This study was approved by the London School of Hygiene & Tropical Medicine Interventions Ethics Committee and the Medical Research Council of Zimbabwe, alongside both the Biomedical Research and Training Institute and Harare City Health review boards. Risk to the foetus was assessed as being relatively minor.


Reviewer #3: The article by Martin et al adds important information regarding the performance of a new point-of-care lateral flow assay for the diagnosis of Neisseria gonorrhoeae (NG-LFA) as a screening test, in pregnant women, from Harare, Zimbabwe. This research is important in extending the NG-LFA assay evaluation in different populations; the initial study of the test indicated high sensitivities and specificities (>90%) in women and men symptomatic for N. gonorrhoeae infection in South Africa. The authors tested 912 pregnant women with the NG-LFA and compared results to the Xpert CT/NG results. The characteristics of participants were noted, and the variables associated with sensitivity and specificity in terms of patient characteristics were also evaluated. The authors ascertained that the sensitivity of the test in this antenatal care population was 65.8%, the specificity was 99.2% and the positive and negative predictive values were78.1% and 98.5%, respectively. They
 
conclude that since one of three N. gonorrhoeae positive cases were not diagnosed using the NG-LFA test, its potential as a screening tool remains to be determined. The authors suggest that it might be of use to diagnose asymptomatic women who would otherwise be untreated, but further studies would be required. They suggest further testing in different populations to assess clinical performance and cost.
The study is skillfully written, and the methods used both to recruit patients and perform testing are solid. The discussion is excellent (lengthy, but a great read), highlighting previous results with the test, differences in study populations and relevant goals for such a test in those populations.

Thank you very much for your comments.

Minor comments:
1.    The most significant criticism of this research would be the lack of good methodology to explain discordant results. Culture, with minimal identification protocols, was attempted but not all specimens were cultured. The authors could provide more information on previous studies regarding the performance of the Xpert test which was used as a gold standard in this analysis.

Thank you for raising this issue. In the limitations section, we do report that the study by Peters et al. had three false negative and two false positive Xpert results. We have now amended the following sentence and reference to show that Xpert is a valid reference test with high sensitivity and specificity:

*“Additionally, we used Xpert as the reference standard. Although a molecular test with high sensitivity and specificity,[31] in the previous evaluation, laboratory-based confirmatory PCR was performed on discordant cases and identified that Xpert had three false negative and two false positive results.[10]” [page 15, lines 508-511]*

We have also added the following sentences to the limitations section to acknowledge the poor yield from culture:

*“Unfortunately, overall yield from culture of NG isolates was relatively poor, with just over a third of individuals with Xpert-positive NG having a positive culture result. Although NG culture was never intended to be the gold standard, it was still less helpful than had been envisioned, particularly in terms of confirming false positive or false negative results. The lack of an on-site incubator to allow for immediate plating, and also variable times between sample collection and transport to the laboratory may have been contributing factors.” [page 15, lines 514-519]*

2.    The authors might consider having Table 1 and Fig 2B as supplementary data.

Thank you for your suggestion. At present, we are happy to keep these in the main paper but also willing to move to supplemental material if reviewers felt strongly. We have however moved table 3 to the supplemental material, and added a new figure 3 (ROC curve).

3.    Xpert NG2 and NG 4, described in Fig 2 is not explained in the methods section.

The following sentence has been added to the final paragraph of the “study procedures” section of the methods:

*“Cycle threshold values for Xpert-positive results were extracted from the GeneXpert output for both NG2 and NG4 targets, which are two separate chromosomal targets for NG, that are amplified as part of results processing [18]. Both NG2 and NG4 targets must be detected for the Xpert result to be reported as positive.” [page 5, lines 180-183]*

4.    The authors could discuss the possibility that the collection of the NG-LFA specimen (the last of three collected) might have affected results regarding bacterial load. Would the load have been reduced with the third specimen taken?

Thank you for your comment. We have added the following sentence:

*“Another consideration that may have contributed to lower bacterial loads was that the NG-LFA specimen was the third swab collected. In contrast, in the previous evaluation by Peters et al. the first swab collected was tested using the NG-LFA.[10]” [page 13, lines 430-432]*

Reviewer #4: This manuscript describes a study to evaluate the performance and acceptability of a lateral flow assay for detecting Neisseria gonorrhoeae (NG-LFA) in pregnant women attending antenatal care (ANC) in Harare, Zimbabwe.  This evaluation was embedded within a prospective study evaluating point-of-care STI screening in pregnant women.  

Although this manuscript is well written with clearly stated objectives and well described study methods, there are serious flaws with several aspects of this study.  Specifically, they are:

1.    Study population:  Study participants were recruited from an ANC clinic regardless of symptom status, ANC visit number, or gestational age.  Screening all pregnant women attending ANC for NG is contrary to current WHO and US CDC guidelines which recommend NG screening only in pregnant women at high risk for STIs.  The authors did not conduct any risk assessment.

This study was approved by the London School of Hygiene & Tropical Medicine Interventions Ethics Committee and the Medical Research Council of Zimbabwe, alongside both the Biomedical Research and Training Institute and Harare City Health review boards. The study protocol submitted to the committees included a dedicated “ethical and safety considerations” section with a specific sub-section “potential risks for pregnant women.”

Furthermore, there have been a plethora of studies in recent years investigating screening for chlamydia, gonorrhoea and trichomoniasis in pregnant women. Most notably, as discussed in the manuscript, the WANTAIM trial in Papua New Guinea found that among pregnant women with NG at enrolment, screening and treatment led to a 53% improvement in prevalence of preterm birth and/or low birth weight. In the WANTAIM study, pregnant women were not risk assessed.

2.    Methods: a) Order of swabs: the swab for the NG-LFA was the last of 3 swabs collected, which may have contributed to the assay's low sensitivity compared to the reference standard assay;  b) Blinding:  the readers of the NG-LFA should have been blinded to the results of the Xpert assay.  The authors reported that, "Mistrust of LFA results was a recurrent theme because of its discrepancies with the Xpert results."

With regards to the order of swabs, we have added the following sentence:

*“Another consideration that may have contributed to lower bacterial loads was that the NG-LFA specimen was the third swab collected. In contrast, in the previous evaluation by Peters et al. the first swab collected was tested using the NG-LFA.[10]” [page 13, lines 430-432]*

With regards to blinding, the intervention team were blinded to the results of the Xpert assay at the time of reading the NG-LFA results, as stated as follows in the methods:

*“Due to the differing analytic times, Xpert results were available after the NG-LFA results, ensuring “blinding” of the study team to NG-LFA interpretation.” [page 4, lines 173-175]*

We agree that ideally those reading the NG-LFA results should have been blinded completely from the Xpert results. However, the parent study was a prospective study evaluating the implementation of point-of-care diagnostics for STIs into antenatal care, and was delivered by a small team, with all tests conducted concurrently. It was therefore not possible for the team to be permanently blinded to the Xpert results as these were required for the team to provide treatment and counselling. We have added the following sentence to the limitations section of the discussion to take account of this, and how it may have influenced the acceptability component of our study:

*“Furthermore, although the intervention team were blinded to Xpert results at the time of reading the NG-LFA, they did subsequently find out the Xpert results, by the nature of the study and having to deliver treatment. This undoubtedly influenced their perceptions of the reliability of the NG-LFA. Of note, if such a test were implemented at scale, concerns regarding accuracy or trust are unlikely to be realised at a local level, if such confirmatory tests are not performed.” [page 15, lines 521-526]*

3.    Sample size: The authors stated that one of the strengths of this study was its large sample size (n=912).  The sample size should have been calculated based on the estimated number of positive and negative samples that would be sufficient to give a 95% confidence interval around the point estimates of sensitivity and specificity.  In this study, only 38 women were positive for NG by the reference standard assay which means that the confidence intervals around the point estimates of sensitivity were very wide. Even the authors admitted that, "it is difficult to draw meaningful conclusions from these observations."

Thank you for raising this point. We have removed sample size as one of the strengths and now state:

*“Importantly, the overall study sample size was not powered for the present evaluation, and was likely underpowered.” [page 15, lines 507-508]*

The statement “it is difficult to draw meaningful conclusions from these observations" was specifically in reference to comparison of sensitivity and specificity across different participant characteristics. Although the confidence intervals for sensitivity and PPV are relatively wide, they still provide very important data regarding the use of the NG-LFA as a screening tool in women. We also demonstrated a very high specificity and NPV wide narrow confidence intervals.

4.    Acceptability of NG-LFA:  The authors made the excuse that, "due to the limited number of interviewees and limited time discussing the NG-LFA within broader interviews, our assessment of acceptability and usability is based on only four viewpoints."  This brings into question whether or not the authors are serious about being able to draw meaningful conclusions from the study results.

The main aim of the present manuscript is the presentation of sensitivity, specificity, PPV and NPV of the NG-LFA among pregnant women attending ANC. Given that we were conducting a broader process evaluation as part of the main study, we opportunistically also included questions focussed on the NG-LFA.

This was limited for two reasons:

i) Given that a 2023 study by de Vos et al. assessed usability of the NG-LFA in South Africa, we felt dedicating significant resources to this question may lead to replication
ii) We had a very small implementing team of only four – given these individuals were those whose viewpoints were most relevant to the implementation of the NG-LFA, we focussed on them.

Nonetheless, we still believe the qualitative data in this study provides important insights into the use of the NG-LFA.
