# Supplementary material for: Evaluation of a novel point-of-care lateral flow assay screening for Neisseria gonorrhoeae infection among pregnant women in Zimbabwe
Source: PLOS Glob Public Health. 2025 Feb 11;5(2):e0003839. doi: 10.1371/journal.pgph.0003839 (PMC11813084; doi:10.1371/journal.pgph.0003839)
Supplement: Attachment — Submitted filename: NG-LFA_response_to_reviewers_26092024.docx [file pgph.0003839.s006.docx]

26^th^ September 2024

Dear Dr Singh,

Thank you for considering our manuscript (PGPH-D-24-01660) for publication in *PLOS Global Public Health*.

We thank the reviewers for their detailed and thoughtful comments. Please find our responses to the editors’ and reviewers’ comments below.

Journal Requirements:

1. We do not publish any copyright or trademark symbols that usually accompany proprietary names, eg (R), (C), or TM (e.g. next to drug or reagent names). Please remove all instances of trademark/copyright symbols throughout the text, including ® on pages 5 and 17.

These instances have been removed.

2. Please review your reference list to ensure that it is complete and correct. If you have cited papers that have been retracted, please include the rationale for doing so in the manuscript text, or remove these references and replace them with relevant current references. Any changes to the reference list should be mentioned in the rebuttal letter that accompanies your revised manuscript. If you need to cite a retracted article, indicate the article’s retracted status in the references list and also include a citation and full reference for the retraction notice.

The reference list has been reviewed, and is complete and correct.

Reviewer comments:

*Reviewer #1:*

There is an urgent need for POCTs for STIs. This article on the use of NG-LFA as a screening tool in pregnant women in Zimbabwe NG highlights important data and recent developments in the field. The paper is well written. The authors have captured both, the advantages as well as limitations of the point-of care test for screening for NG in setting which follows the syndromic management of STIs. This assay has already demonstrated a high sensitivity and specificity (>90%) in symptomatic individuals. It was only logical to next evaluate its performance & acceptability as a screening tool.

Thank you for your comments.

I have minor comments for the authors :

1. In the Laboratory procedures, the authors have not carried out confirmatory testing for NG. The tests mentioned only provide presumptive identification of NG.

We have added the word “presumptively” to the methods to reflect this, as follows:

*“NG was presumptively identified by the presence of all of…”* [page 5, line 194]

2. The false-negative results by NG-LFA were seen basically in samples that had a low bacterial load. We do expect a low load in asymptomatic patients. Subsequently, it is mentioned that there was no difference in Ct values between those with and without symptoms, for both NG2 (median 26.3 vs 26.2; p = 0.90) and NG4 (median 26.3 vs 26.3; p=0.79) targets. Please explain.

We feel there are several potential reasons for this. Firstly, the number of participants with symptoms was low and so it difficult to draw strong conclusions from this data. Secondly, participants who report the presence of symptoms when asked are likely different to individuals who have sought care for their symptoms, and are a unique group in this sense. This, alongside the common physiological changes to vaginal discharge during pregnancy, make interpretation of this data difficult and we would be wary of drawing any conclusions.

Finally, in a study by van der Veer et al. (2020), although they found an association between presence of symptoms and bacterial load in male urine samples, there was no such association for vaginal samples. As a result, the lack of association in our study is not without precedent.

We have amended the following paragraph as follows to reflect this:

*“We compared sensitivity and specificity across a range of participant characteristics. However, due to the small sample sizes for sensitivity calculations, it is difficult to draw meaningful conclusions from these observations. Caution must also be exercised when comparing these findings to the study among symptomatic individuals in South Africa.[10] Individuals attending clinic because they have symptoms are likely very different to those attending for routine ANC and reporting symptoms when specifically asked. Firstly, physiological changes to vaginal discharge are common in pregnancy. Furthermore, the former’s symptoms are more likely to be of a higher severity or duration such that the individual has sought care. For these reasons we are also wary of interpreting the lack of difference in Ct values between participants with and without symptoms.”* [page 14, lines 466-474]

3. Gonococcal culture: Of the 13 discordant results by NG-LFA, 2 (15.4%) were culture positive. This is a matter of serious concern. What is the limit of detection of NG-LFA ?

Thank you for your comment. In prior laboratory evaluations, the limit of detection with reference NG strains was 5 × 10³ colony forming units per mL. Of note, we have noted an error in the manuscript, whereby it was in fact three discordant results that were culture positive rather than two (this has now been rectified).

One consideration is that for culture, cervical swabs were taken compared to the vaginal swabs used for the NG-LFA. We would expect bacterial load to be much higher at the cervix. The cycle threshold values somewhat concur with this for two of the three isolates, with Ct values > 30, as follows:

Isolate 1 – NG2 30.9, NG4 30.7

Isolate 2 – NG2 37.4, NG4 37.8

Isolate 3 – NG2 25.3, NG4 25.1

We have added the following sentence to the discussion to draw the readers’ attention to this:

*“Of note, there were three false negative results that were culture positive, indicative of viability. This discrepancy may due to the different sampling sites for the NG-LFA and for culture. Whereas swabs for NG-LFA were vaginal, swabs for culture were taken from the cervix, where both bacterial load and organism viability is likely to be higher.”* [page 14, lines 460-464]

4. Some issues can be explained by the fact that the third swab was used for NG-LFA.

Thank you for your comment. We have already included this as a potential factor in the discussion, as follows:

*“Another consideration that may have contributed to lower bacterial loads was that the NG-LFA specimen was the third swab collected. In contrast, in the previous evaluation by Peters et al. the first swab collected was tested using the NG-LFA.[10]”* [page 13, lines 433-435]

As such, we have not amended the text further.

5. One third of pregnant women were missed by NG-LFA. This has important implications particularly, involving the neonate. No doubt, we need screening tools with better sensitivity and more research is needed in this direction.

Thank you very much for your comments. We completely agree, and feel that our concluding paragraph already aligns with these important points.

*Reviewer #2:*

1. The study is part of larger approved size for which the sample size calculation was done. Hence, the sample size seems likely underpowered for the present study. Even though drawing meaningful interpretations from current sample size is difficult; the results available for the current sample size are well presented and interpreted highlighting the existing limitations.

Thank you for your comments.

2. On its own the study is more suitable to be presented as a pilot work, based upon whose results a comprehensive study to evaluate the LFA may be planned including adequate sample size for evaluating both the performance characteristics and operational characteristics of the LFA considering:
   a. Both symptomatic/ asymptomatic participants
   b. High risk population/ general population
   c. Types of samples including (vaginal swab/ urine/ rectal swab/oral swab/ cervical swabs etc.)
   d. self collected/ clinician collected samples

This will present a correct picture of the utility of the LFA especially in resource limited settings where self collected samples and urine samples may be more relevant.

We acknowledge the limitation of the study sample size in our strengths and limitations section, as follows:

“Importantly, the overall study sample size was not powered for the present evaluation, and was likely underpowered.” [page 15, lines 517 – 518]

We also agree that more studies are required covering a range of populations, sample types and sample collection methods. We have amended the following statement to reflect this:

*“More studies are needed to assess the NG-LFA in other settings and populations, across different sample types and sample collection methods, to further explore the relationship between NG-LFA positivity, bacterial load and infection viability, and to understand the clinical implications of these low bacterial load infections.”* [page 16, lines 546-549]

3. Nothing is mentioned regarding the financial implications and the time to result for the LFA when the operational characteristics are being evaluated in terms of it meeting the "Target Product Profiles" defined for PoCT for Neisseria gonorrhoeae by the WHO Consultation on PoCT.

Thank you for comment and suggestions. At present, our article is quite diverse in that it covers clinical performance of the NG-LFA, laboratory data regarding culture and cycle threshold values, and an assessment of acceptability and usability from the perspective of users.

Given that our discussion is already very lengthy, we are reticent to widen it further by discussing whether the NG-LFA meets the target product profile characteristics set out by WHO and FIND, and particularly the financial implications of this. Although the target product profiles discussed by Ferreyra et al. (2020) do include mention of screening of “high-risk populations”, they are much more focussed on the use of a point-of-care test for symptomatic individuals.

Additionally, how the NG-LFA fits in with the target profile characteristics is discussed in more detail in the following two publications, and including it risks replication:

Gleeson B, Piton J, Mazzola L, et al. Development of a Novel Fluorescent-Based Lateral Flow Assay for the Detection of Neisseria gonorrhoeae at the Point of Care. Sex Transm Dis. 2024;51(3):186-191. doi:10.1097/OLQ.0000000000001913

Peters RPH, Klausner JD, Mazzola L, et al. Novel lateral flow assay for point-of-care detection of Neisseria gonorrhoeae infection in syndromic management settings: a cross-sectional performance evaluation. Lancet. 2024;403(10427):657-664. doi:10.1016/S0140-6736(23)02240-7

4. An important aspect to be re-looked for the LFA would be strengthening the quality assurance of the reader which may give human/ technical errors. (four false negatives were identified within 8 days at a particular site).

Yes, we agree that ensuring quality assurance is essential for the NG-LFA going forward. We have amended the following paragraph of the discussion to note this:

*“Other possibilities for false negative results include human error or issues with the readers. The four readers used in the study were prototype models and could have had technical issues. There were four false negative results within eight days at one site in August 2023. However, prior to re-training, practice tests using positive and negative control samples were correctly identified by both readers at the site. As no obvious errors could be identified retrospectively, and the relatively high Ct values among the false negative cases in this period (NG4 Ct > 30 for three out of four cases), this may have been a random increase in the frequency of false negative cases. There was also no difference in sensitivity between sites and readers, but confidence intervals were wide. Strengthening and ensuring quality assurance of the reader will be particularly important prior to commercialisation of the NG-LFA.”* [page 13, lines 424-433]

5. All these comments become even more significant considering the fact that 80-90% of STI burden exists in developing world where the PoCTS become most relevant due to lack of skilled manpower and infrastructure. These sites may lack the basic facility for sample collection and processing (reader use) making ease-of-use (operational characteristics) most important when using the LFA.

Thank you very much – we completely agree. We have amended the following paragraph to highlight how the ease-of-use of the NG-LFA that we found is particularly beneficial for resource-limited settings, as follows:

*“The NG-LFA was reported to be acceptable, easy-to-use, easy-to-interpret, and portable, concurrent with the SUS scores. These characteristics are particularly important when considering implementation in settings with limited infrastructure and where retention of a skilled workforce is challenging.”* [page 15, lines 492-494]
